# Impaired skeletal muscle microcirculation in systemic sclerosis

**DOI:** 10.1186/ar4047

**Published:** 2012-10-04

**Authors:** Sasan Partovi, Anja-Carina Schulte, Markus Aschwanden, Daniel Staub, Daniela Benz, Stephan Imfeld, Björn Jacobi, Pavel Broz, Kurt A Jäger, Martin Takes, Rolf W Huegli, Deniz Bilecen, Ulrich A Walker

**Affiliations:** 1 Department of Radiology and Nuclear Medicine, University Hospital Bruderholz, Kantonsspital Bruderholz, CH-4101 Bruderholz, Switzerland; 2 Department of Angiology, University Hospital Basel, Petersgraben 4, CH 4031 Basel, Switzerland; 3 Department of Rheumatology, University Hospital Basel, Felix Platter-Spital, Burgfelderstrasse 101, CH 4012 Basel, Switzerland

**Keywords:** Systemic sclerosis, skeletal muscle, vasculopathy, magnetic resonance imaging

## Abstract

**Introduction:**

Muscle symptoms in systemic sclerosis (SSc) may originate from altered skeletal muscle microcirculation, which can be investigated by means of blood oxygenation level dependent (BOLD) magnetic resonance imaging (MRI).

**Methods:**

After ethics committee approval and written consent, 11 consecutive SSc patients (5 men, mean age 52.6 years, mean SSc disease duration 5.4 years) and 12 healthy volunteers (4 men, mean age 45.1 years) were included. Subjects with peripheral arterial occlusive disease were excluded. BOLD MRI was performed on calf muscles during cuff-induced ischemia and reactive hyperemia, using a 3-T whole-body scanner (Verio, Siemens, Erlangen, Germany) and fat-suppressed single-short multi-echo echo planar imaging (EPI) with four different effective echo times. Muscle BOLD signal time courses were obtained for gastrocnemius and soleus muscles: minimal hemoglobin oxygen saturation (T2*_min_) and maximal T2* values (T2*_max_), time to T2* peak (TTP), and slopes of oxygen normalization after T2* peaking.

**Results:**

The vast majority of SSc patients lacked skeletal muscle atrophy, weakness or serum creatine kinase elevation. Nevertheless, more intense oxygen desaturation during ischemia was observed in calf muscles of SSc patients (mean T2*_min _-15.0%), compared with controls (-9.1%, P = 0.02). SSc patients also had impaired oxygenation during hyperemia (median T2*_max _9.2% vs. 20.1%, respectively, P = 0.007). The slope of muscle oxygen normalization was significantly less steep and prolonged (TTP) in SSc patients (P<0.001 for both). Similar differences were found at a separate analysis of gastrocnemius and soleus muscles, with most pronounced impairment in the gastrocnemius.

**Conclusions:**

BOLD MRI demonstrates a significant impairment of skeletal muscle microcirculation in SSc.

## Introduction

Systemic sclerosis (SSc) is a connective tissue disorder in which vascular alterations and endothelial damage are prominent and lead to progressive and widespread microangiopathy with dysfunction of various organs [1,2]. The impaired microcirculation may become clinically apparent as Raynaud's phenomenon, digital ulcers (DU), pulmonary hypertension, or renal crisis [1,2]. About one third of SSc patients complain of muscle weakness, 15% have objective muscle atrophy, and 10% an elevated serum creatine kinase (CK) [3]. The exact pathogenesis of the muscle involvement is unknown, but muscle biopsies in SSc patients have demonstrated increased fibrosis of the perimysium and epimysium [4-6], intimal proliferation of the larger endomysial and perimysial vessels, perivascular infiltrates or muscle necrosis [4-8]. It is however unclear, if there is a functional impairment of skeletal muscle microcirculation in SSc patients.

Blood oxygenation level-dependent (BOLD) magnetic resonance imaging (MRI) has been shown to be a valuable tool for the assessment of skeletal muscle microcirculation [9-11]. Derived from functional brain MRI studies, this technique relies on the physiologic magnetic properties of hemoglobin and is therefore independent from exogenous contrast agents [12]. Hemoglobin iron changes its spin state from diamagnetic low-spin in the oxygenated state to paramagnetic high-spin in the deoxygenated state [13]. This causes local magnetic field distortions in the surrounding tissue, which results in dephasing of the proton signal, consecutively leading to a signal decay with increasing intravascular deoxyhemoglobin content [12]. Gradient echo (GE) MR sequences emphasize this effect, leading to an increase of the apparent transverse relaxation rate (1/T2*) and a decrease of T2*[14]. Though being mainly determined by the oxygen saturation in muscle microcirculation, BOLD signal also depends on blood volume, hematocrit and inflow [10,15]. By provoking changes in the local muscle oxyhemoglobin concentration via ischemia, reactive hyperemia, drugs or muscle exercise, BOLD imaging can be used to assess physiologic and pathologic alterations of micro- and macrovascular pathologies [10,16-19].

These properties render muscle BOLD imaging a promising method for the assessment of the microangiopathic component in the muscular symptoms of SSc patients. Despite a high prevalence of muscular complaints and findings, a systematic evaluation of muscle microperfusion has not yet been performed. The purpose of this study was therefore to analyze the microcirculation of two different calf muscle groups in SSc patients using an ischemia/ reactive hyperemia paradigm, and to compare the T2* time courses of SSc patients with those of healthy volunteers.

## Materials and methods

### Subjects

The study protocol was approved by the institutional review board and the local ethics committee. Consecutive patients with SSc, as defined by the American College of Rheumatology (ACR) [20] and healthy volunteers were recruited at our institution. All subjects were required to be older than 18 years of age, normotensive, to have a normal peripheral pulse status and ankle-brachial indexes (ABI) ≥ 0.9. Exclusion criteria were general contraindications to MRI such as cardiac pacemakers, ferromagnetic implants, immobility, pregnancy and claustrophobia. All subjects gave written informed consent according to the declaration of Helsinki.

### Muscle BOLD paradigm

**Figure 1:**
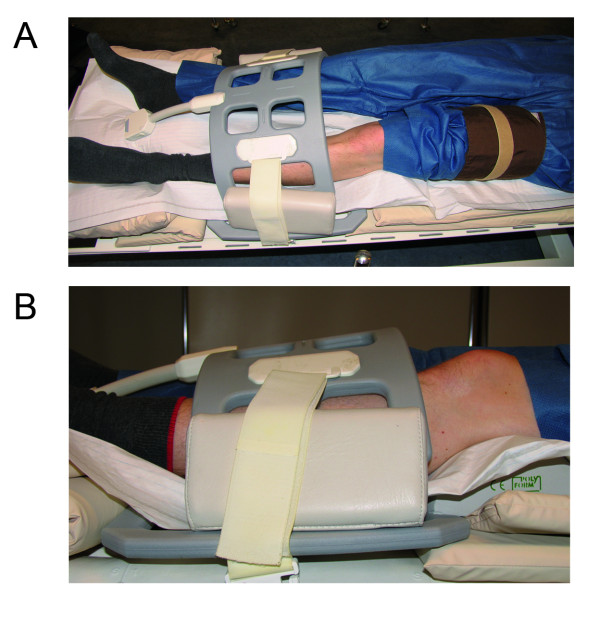
**Imaging setup of the ischemia/reactive hyperemia paradigm conducted with a conventional leg sphygmomanometer wrapped around the middle of the thigh and flexible array coils**. To prevent compression of the calf muscles the lower leg is supported at knee and foot level.

Subjects were placed supine with feet first within the magnet bore and had to rest at least 5 minutes before starting the examination to minimize the degree of venous filling of the calf (Figure 1). A conventional leg-sphygmomanometer cuff was fixed at mid-thigh level. Ischemia of the leg was achieved by fast manual inflation of the cuff to an end occlusion pressure of 50 mmHg above the individual brachial systolic blood pressure. After 180 s of cuff compression, the cuff was released quickly by opening the air valve. Muscle BOLD imaging was performed during the first 60 s in the resting state (baseline), the following 180 s of ischemia and during reactive hyperemia until recovery for a further 400 s. Overall, 320 consecutive scans were performed within a total acquisition time of 640 s.

### MRI technique

**Figure 2:**
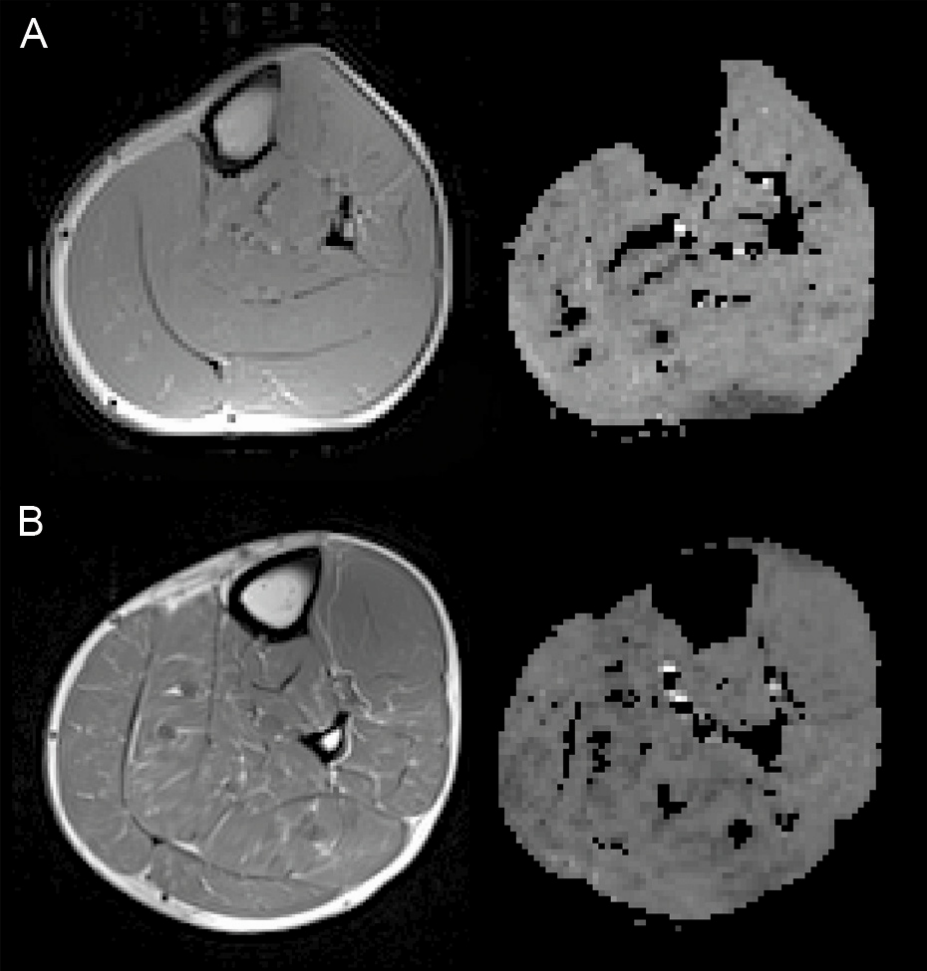
T1 images and T2* maps of the upper left calf region from a healthy volunteer and a patient with systemic sclerosis (SSc). T1 images are shown on the left and T2* maps on the right. (**A**) Healthy volunteer. (**B**) SSc patient. T1 images were correlated for anatomical reference with the respective T2* maps.

All muscle BOLD MRI measurements were performed on a 3-T whole-body scanner (Verio, Siemens Medical Solutions, Erlangen, Germany) [11]. A fat-suppressed, T2*-weighted, single, short multi-echo echo planar imaging (EPI) sequence was used. Four axial slices (slice thickness 5 mm, gap 5 mm) were positioned in the upper left calf at maximum diameter. Imaging parameters were as described previously [11]. EPI images were supplemented with anatomical reference images of the four corresponding slices using a T1-weighted, spin-echo sequence (Figure 2).

**Figure 3:**
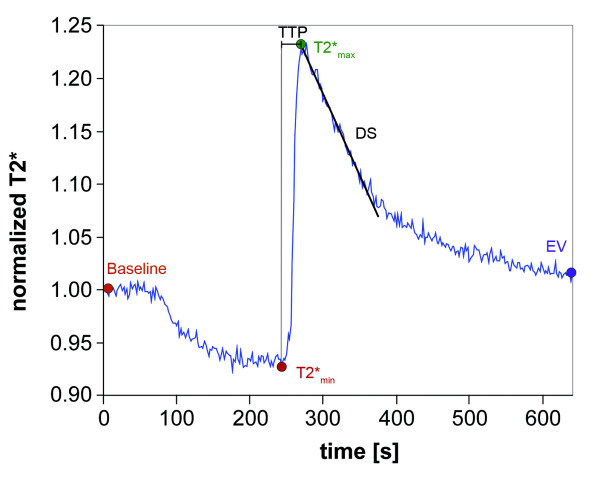
Key parameters of a typical blood oxygenation level-dependent (BOLD) T2* signal time course in a healthy volunteer. T2* is a magnetic resonance imaging signal.

T2* time courses were obtained from rectangular regions-of-interest (ROIs) within the soleus and gastrocnemius muscle using BrainVoyager (Brain Innovation B.V., Maastricht, Netherlands). The ROIs were chosen to exclude pixels of large vessels. All T2* time-courses were normalized with respect to baseline and averaged over the four acquired slices in each subject. Five curve parameters for the mean T2* time course of every subject were determined (Figure 3): 1) T2*_min_: minimum T2* value during ischemia relative to baseline; 2) T2*_max_: hyperemia peak-value of T2* relative to baseline; 3) time to T2* peak (TTP): time in seconds between cuff deflation and T2*_max_; 4) declining slope (DS): T2* gradient between T2*_max _and 60 s afterwards (DS_60s_) and between T2*_max _and 120 s afterwards (DS_120s_); 5) T2* end-value (EV): average T2* during the last 10 s of the 640 s measurement, relative to baseline. T2*_min _is a BOLD parameters characteristic of the microcirculatory network during ischemia, whereas T2*_max _, TTP, DS and EV are key parameters of the hyperemia phase.

### Statistical analysis

For each muscle, mean values of the curve parameters were computed by averaging over all subjects belonging to the same study group. In addition, mean time courses of the two groups were calculated separately from individual T2* time courses in the soleus and gastrocnemius muscle. Statistical analysis of the curve parameters was performed with the Sigma-Plot vs. 12.2 statistical package (Systat Software Inc, San Jose, CA, USA). Curve parameters were compared between study groups by means of two-sided, unpaired Student's *t*-tests or Wilcoxon's rank sum tests after Kolmogorov-Smirnov testing for normality. *P*-values lower than 0.05 were considered statistically significant. Relationships between clinical characteristics and muscle BOLD parameters were computed as Spearman's rank-order or Pearson's moment correlation, as appropriate.

## Results

Twelve healthy volunteers (four male) and eleven SSc patients (five male) were recruited. The mean age of the SSc patients was 52.6 years, (SD 10.0) and the mean age of the volunteers was 45.1 years (SD 13.1). There was no statistical age difference between the study groups. None of the study participants had a known malignancy. The average ankle brachial index was 1.1 (SD 0.08) in the healthy volunteers and 1.16 (SD 0.08) in the SSc patients (*P*= 0.15). The mean body mass index (BMI) was 25.8 (SD 3.7) in the SSc patients and was similar to that in the healthy controls (mean 23.6, SD 2.7, *P *= 0.13). None of the subjects had diabetes mellitus. Only one of the subjects, a female SSc patient, had anemia (hemoglobin concentration 118 g/L blood).

Most SSc patients lacked muscle symptoms (Table 1). One male SSc patient however, had muscle atrophy with symmetric proximal and distal muscle weakness; his serum CK (normal value <190 U/L) was 1570 U/L. Gastrocnemius muscle biopsy in this patient revealed scattered necrosis in the absence of inflammation or centralized myonuclei. In a second male SSc patient, the serum CK was slightly elevated (218 U/L). Five SSc patients had a history DU; of these two had DU at the time of imaging. None of the SSc patients had pulmonary arterial hypertension, or a history of renal crisis. Five patients were treated with prednisone at the time of imaging, only one of these patients was treated with a daily dose above 7.5 mg (15 mg). Seven patients received a second immunosuppressive agent and no patient was treated with iloprost or a phosphodiesterase-5 inhibitor; one patient received bosentan.

**Table 1:** Clinical characteristics of eleven patients with systemic sclerosis undergoing blood oxygenation level-dependent MRI of the calf muscles

Characteristic	Value
**SSc disease characteristics**	
SSc duration by first non-Raynaud's symptom, mean (years)	5.4, SD 5.0
SSc duration by onset of Raynaud's phenomenon, mean (years)	6.8, SD 6.2
Diffuse cutaneous SSc, n	3
Limited cutaneous SSc, n	5
Other SSc, n	3
Modified Rodnan skin score, median	5, range 1-26
Antinuclear autoantibody positive, n	11
Anti-centromere autoantibodies positive, n	3
Anti-topoisomerase autoantibodies positive, n	5
PM-Scl autoantibodies positive, n	1
Erythrocyte sedimentation rate, mean (mm)	14, SD 12
**Muscle parameters**	
Serum creatine kinase elevation, n	2
Muscle atrophy, n	1
Muscle weakness on manual muscle testing, n	1
Six-minute walk test distance, mean (meters)	449, SD 68
Cardiopulmonary function	
Systolic blood pressure, mean (mmHg)	127, SD 17
Diastolic blood pressure, mean (mmHg)	78, SD 11
Systolic pulmonary arterial pressure by echocardiography, mean (mmHg)	23.7, SD 4.4
Forced vital capacity, mean % of normal	91.4, SD 18.6
Diffusing capacity of the lung for carbon monoxide, mean % of normal	73.1, SD 26.3
**SSc therapy**	
Patients on prednisone, n	5
Patients on methotrexate, azathioprine, mycophenolate, or cyclophosphamide, n	7

SSc, systemic sclerosis; BOLD, blood oxygenation level-dependent; MRI, magnetic resonance imaging; n, number of patients.

**Figure 4:**
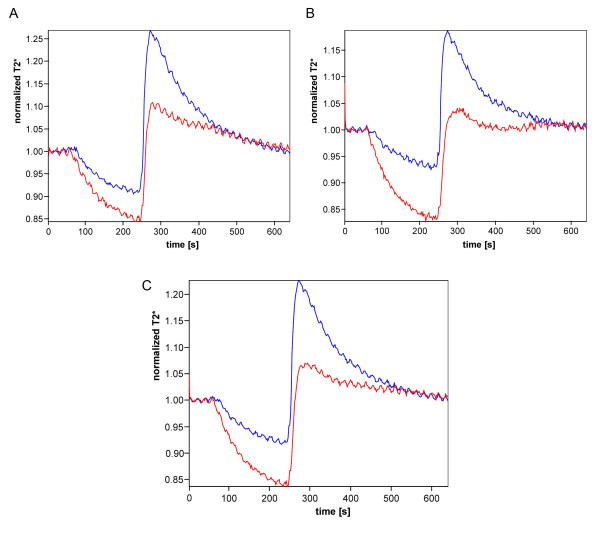
**Muscle blood oxygenation level-dependent (BOLD) time courses in patients with scleroderma and healthy controls**. Time courses for patients with scleroderma are shown in red and for healthy controls in blue in (**A**) the soleus and (**B**) the gastrocnemius muscle. (**C**) Mean time courses over both muscle groups. T2* is a magnetic resonance imaging signal.

Muscle BOLD MRI measurements were performed successfully and were well-tolerated in all subjects. The size of the ROIs in the soleus was 121 pixels (SD 36), corresponding to a mean value of 4.8 cm^2 ^(SD 1.4), and in the gastrocnemius 152 pixels (SD 35), corresponding to a mean value of 6.1 cm^2 ^(SD 1.4). Figure 4 depicts the mean T2* time courses of the soleus (Figure 4a) and gastrocnemius muscles (Figure 4b) and the mean of both muscles (Figure 4c) in SSc patients (red) and healthy volunteers (blue). During ischemia, the T2* value continuously decreased in both study groups. However, in the patient group, the mean muscle BOLD T2* value in both calf muscles dropped faster and to a significantly lower value (T2*_min _-15.0%, SD 7.3), than in the control group (-9.1%, SD 3.5, *P *= 0.021) (Table 2).

**Table 2:** Calf muscle blood oxygenation level-dependent (BOLD) key parameters of all eleven patients with systemic sclerosis (SSc) and twelve volunteers

	Volunteers	SSc patients	*P*-value
T2*_min _(%)			
Soleus, mean (SD)	-10.3 (4.0)	-13.4 (6.9)	0.20
Gastrocnemius, mean (SD)	-8.0 (4.6)	-18.0 (9.9)	0.005
Both, mean (SD)	-9.1 (3.5)	-15.0 (7.3)	0.021
T2*_max _(%)			
Soleus, median (IQR)	26.4 (20.0, 30.6)	15.7 (6.5, 21.9)	< 0.007
Gastrocnemius, median (IQR)	17.2 (9.4, 32.0)	4.3 (0.1, 6.4)	0.002
Both, median (IQR)	20.1 (17.0, 29.6)	9.2 (4.4, 17.5)	0.007
**TTP (s)**			
Soleus, median (IQR)	32.0 (30.0, 35.5)	38.0 (34.0, 54.0)	0.037
Gastrocnemius, median (IQR)	34.0 (27.5, 37.3)	42.0 (38.0, 50.0)	0.002
Both, median (IQR)	33.5 (29.5, 36.5)	39.0 (35.0, 56.0)	0.026
DS_60 s _*10-4 (s^-1^)			
Soleus, mean (SD)	-17.9 (4.9)	-10.3 (5.7)	0.002
Gastrocnemius, mean (SD)	-14.7 (6.1)	-6.6 (7.0)	< 0.001
Both, mean (SD)	-16.5 (4.6)	-7.3 (4.9)	< 0.001
DS_120 s _*10-4 (s^-1^)			
Soleus, mean (SD)	-14.8 (3.3)	-6.2 (4.4)	< 0.001
Gastrocnemius, mean (SD)	-11.7 (5.6)	-4.5 (4.9)	0.004
Both, mean (SD)	-13.7 (4.1)	-4.6 (3.6)	<0.001
**EV (%)**			
Soleus mean (SD)	-0.3 (3.9)	1.0 (4.4)	0.45
Gastrocnemius, mean (SD)	0.8 ± 3.3	-0.3 (3.4)	0.41
Both, mean (SD)	-0.7 (4.2)	-0.3 (2.7)	0.77

Parameters were obtained for the soleus and gastrocnemius muscle. Normally distributed parameters are provided as means with SD, non-normally distributed parameters are provided as medians with interquartile ranges (IQR). T2*_min_, ischemic minimum of T2*; T2*_max_, T2* peak value during reactive hyperemia; TTP, time to peak; DS, declining slope after hyperemia peaking; EV, T2* end value. T2*_min_, T2*_max _and EV were calculated relative to baseline. DS_60s _and DS_120s _represent T2*declining slopes between T2*_max _and 60 s or 120 s afterwards.

T2* quickly increased in both groups after cuff deflation, resulting in a hyperemia peak that was significantly reduced in SSc patients (median T2*_max _9.2%, interquartile range (IQR) 4.4, 17.5), compared to the control group (20.1%, IQR 17.0, 29.6, *P *= 0.007). The median TTP was 39.0 s (IQR 35.0, 56.0) in the patient group and 33.5 s (IQR 29.5, 36.5) in the healthy volunteers (*P *= 0.026). After T2* peaking, the muscle BOLD signal decay was faster in the control group during the first 60 s than during the next 60 s in all muscles examined. In both time periods, DS values were significantly shorter in the healthy volunteers than in the SSc patients (Table 2). Finally, in both groups the T2* calf muscle time course returned to approximately baseline values with a EV mean value of -0.3, SD 2.7% in the SSc group and -0.7, SD 4.2% in the control group (*P*= 0.77).

Within the SSc patient group, neither the severity of skin involvement in terms of the modified Rodnan skin score (mRSS), nor patient age, disease duration, or the result of the six-minute walk test were correlated with any BOLD parameter. There were also no significant differences in BOLD parameters with respect to limited and diffuse cutaneous SSc subsets, or autoantibody subgroups.

When comparing T2* time courses between the soleus and the gastrocnemius muscles in healthy volunteers, the more oxidative soleus muscle had a slightly better oxygenation during reactive hyperemia (T2*_max_), than the more glycolytic gastrocnemius muscle (*P *= 0.016, paired *t*-test), and a trend towards a faster return to baseline in terms of DS values. When comparing the muscle BOLD MRI curves of the soleus and gastrocnemius in the SSc population, the oxygenation of the gastrocnemius muscle during ischemia and reactive hyperemia appeared to be more impaired than that of the soleus muscle (Table 2, Figures 4a and 4b). Statistical significance was observed when T2*_max _values were compared between soleus and gastrocnemius muscles in SSc (*P *= 0.010, paired *t*-test).

## Discussion

We investigated skeletal muscle microcirculation in SSc patients using skeletal muscle BOLD MRI of the calf. Muscle BOLD time courses revealed a pronounced impairment of muscle microcirculation in SSc patients compared to healthy volunteers. The overall characteristics of the muscle T2* time course during ischemia and reactive hyperemia in the group of healthy volunteers were similar to those already published [10,11,15,17].

During the ischemia phase, T2* dropped to significantly lower T2*_min _values in the SSc group compared with the control group. This finding could be explained by a reduced oxygen reservoir in the microcirculation due to capillary loss or obliteration. After cuff deflation, SSc patients showed a significant reduction of T2*_max_, a marked prolongation of TTP, and decreased DS values compared to healthy volunteers. These effects could also be explained by a reduction of blood flow in the microcirculation due to precapillary occlusion or a reduction of capillary density, that is, structural vasculopathy. A reduced vasodilatation reserve or preponderance of vasoconstrictor stimuli (that is, functional vasculopathy) could also be responsible for the observed alterations. Certainly, several of the listed phenomena could concurrently contribute to these findings. Irrespective of the mechanisms, the observed changes in the curve characteristics are most probably attributable to local alterations of microcirculation, a known hallmark of SSc in other organs [1,2].

Concerning intermuscular differences, BOLD signal alterations between SSc patients and healthy volunteers tended to be more pronounced in the gastrocnemius muscle when compared with the soleus muscle. Soleus muscle predominantly consists of slow twitch oxidative muscle fibers, whereas the gastrocnemius muscle is mostly composed of fast twitch glycolytic fibers [15]. The more pronounced impairment of the oxidative soleus muscle in SSc than that of the more glycolytic gastrocnemius muscle may be explained by differences in the blood supply. The gastrocnemius is commonly supplied by a single artery that divides into branches, whereas at least five separate arteries successively enter the soleus [21]. Therefore the blood supply to the soleus may be more vulnerable to obliteration than that of the gastrocnemius.

In several rheumatic disorders, a macrovascular involvement has been postulated; however, the frequency of atherosclerosis and its extent in SSc remain controversial [22-24]. As we excluded subjects with pre-existing peripheral artery occlusive disease (PAOD) from our study, and the time courses of SSc patients differ substantially from those of patients with PAOD during ischemia, our results primarily implicate small vessel disease as the origin of the detected BOLD signal alterations in skeletal muscle. A recent analysis of skeletal muscle involvement in SSc patients via 99mTc sestamibi scintigraphy similarly revealed significant impairment of muscle perfusion compared with healthy volunteers [25].

Our study has several limitations. First, our study only includes a relatively small and heterogeneous number of patients and volunteers. However, even in this small collection, highly significant BOLD key parameter alterations could be demonstrated. Second, there were some imbalances in age and BMI between the study groups. Earlier studies demonstrated that BOLD time courses in calf muscles are age- and BMI-dependent [11,17]. As age and BMI differences were considerably larger in these aforementioned studies (30 to 43 years), the effect on our results is likely to be rather small. T2*min and T2*max decrease in older persons, thus we might underestimate the alterations in SSc in our study.

Although symptoms and findings indicative of muscular involvement are frequent in SSc [3], the SSc-related myopathy lacks a universally accepted gold standard for its diagnosis and is heterogeneous. Muscle weakness in SSc may also arise from extramuscular organ involvement such as cardiac, cutaneous, or pulmonary complications, or gastrointestinal malassimilation. Serum CK measurements are not a sensitive diagnostic tool, because a substantial number of SSc patients with objective muscle weakness or abnormal muscle histology have normal CK levels [6,26]. Thus, BOLD MRI may become a valuable aid in the differential diagnosis of muscle weakness, myalgia and fatigue of SSc patients.

Most of the consecutive SSc patients included in this study had a functional impairment of muscle microcirculation despite a relatively short SSc disease duration and the absence of muscle symptoms and findings. This suggests that skeletal muscle microangiopathy may be an early and prevalent SSc characteristic, although only a minority of patients with SSc-related myopathy has biopsy-proven muscle microangiopathy [27]. Further studies correlating muscle BOLD MRI findings with clinical and histopathological data might be helpful in discriminating the influence of different mechanisms on the alternations in the T2* time courses in SSc patients and disease subgroups. Future studies of BOLD MRI may determine if the microcirculatory impairment is reversible, or if it indicates irreversible muscle damage. The cuff compression paradigm that is used allows the examination of forearm, hand, calf, and foot muscles and thus may be used for the determination of disease state and progression. As Raynaud's phenomenon typically appears on the digits and finger tip ulceration is a common complication in SSc patients, it will be interesting to investigate if BOLD MRI changes are more pronounced in distal than proximal limb muscles and whether they are reversible or preventable with therapeutic agents.

## Conclusions

We provide the first *in-vivo *evidence for an impaired skeletal muscle microcirculation in SSc by performing skeletal muscle BOLD MRI. Muscle BOLD MRI represents a suitable and non-invasive imaging method for SSc-associated vasculopathy. Further studies are warranted to get additional insights into the mechanisms underlying muscle BOLD signal alterations in patients with SSc. This method provides a non-invasive diagnostic tool in the assessment of muscle involvement in SSc, and perhaps also other rheumatic conditions such as connective tissue diseases and small vessel vasculitis.

## Abbreviations

ACR: American College of Rheumatology; BMI: body mass index; BOLD: blood oxygenation level-dependent; CK: creatinine kinase; DS: declining slope; DU: digital ulcer; EPI: echo planar imaging; EV: end value; GE: gradient echo; IQR: interquartile range; MRI: magnetic resonance imaging; PAOD: peripheral artery occlusive disease; ROI: region of interest; mRSS: modified Rodnan skin score; SSc: systemic sclerosis; T2*: T2 star (an MRI signal); T2*min: minimal hemoglobin oxygen saturation; T2*max: maximal hemoglobin oxygen saturation; TTP: time to T2* peak.

## Competing interests

There are no other financial interests of any of the authors which could create a potential conflict of interest or the appearance of a conflict of interest with regard to the work.

## Authors' contributions

All authors have made substantial intellectual contributions to the content of this manuscript in different categories. In detail the participation of each author is as follows: UAW as senior author, DB and KAJ guarantee study integrity. The overall study concept and design was created by SP, UAW and DB. Acquisition of data was done by MA, DS, DB, PB and MT. Data analysis and interpretation was performed by SP and A-CS. The literature search was performed by SP, UAW, A-CS, SI and BJ. A-CS and SP performed the statistical analysis. SP and UAW drafted the manuscript. The manuscript was first revised critically by A-CS, MA, DS, RWH, BJ and DB. All authors edited the manuscript and gave their approval to the final version.
